# Recapitulating intracavitary brachytherapy in cervical cancer patients during the COVID-19 pandemic: a viewpoint

**DOI:** 10.2217/fon-2020-0588

**Published:** 2020-08-10

**Authors:** Divyesh Kumar, Treshita Dey

**Affiliations:** ^1^Department of Radiotherapy & Oncology, PGIMER, Chandigarh 160012, India

The novel coronavirus disease 2019 (COVID-19) pandemic has impacted the healthcare sector worldwide. The WHO declared it a public health emergency on March 11th, 2020. The respiratory virus, SARS-CoV-2, is transmitted via direct contact or droplets from an infected person with a secondary attack rate of more than 90%. Hence, social distancing and containment along with proper hygiene have evolved as the key steps to flatten the transmission curve.

Cancer care hangs on a fine scale of balance amidst the COVID-19 pandemic. Worse survival in oncology patients has been reported due to their compromised immunity, which may be related to either the nature of their disease or their treatment. The risk of death related to COVID-19 infection among patients with cancer is also substantially higher, particularly in those aged over 60 years and patients with pre-existing pulmonary disease [1]. Although the evidence to this is preliminary [2], the situation has put the oncologists in great jeopardy and has led them to triage patients based on essential treatment services and optimal outcomes. During the COVID-19 crisis, delaying treatment might remove the only possible chance to cure the patient, while continuing treatment can weaken their immune system and make them more prone to infection. Thus, striking a balance between cancer treatment and delay, along with the probable risks of acquiring infection during this period, is a matter of concern and should be thoroughly discussed with patients and their caregivers. Furthermore, overall treatment time (OTT) has an important role in determining treatment outcome in patients undergoing radiotherapy. Cervical cancer is one of the leading causes of malignancy in females, and prolonging the OTT negatively affects the outcome. Hence treatment interruption should be avoided whenever possible, even during the pandemic.

In this acute outbreak, our health-related workforce has restricted capacity, but a rational and strategic reorganization has allowed us to keep routine cancer care functional. To minimize the burden against the background of the current pandemic, alternative treatment protocols or recommendations, such as using altered fractionation and avoiding general anesthesia (GA) for brachytherapy, are the need of the hour.

Though many centers worldwide have documented on the role of hypofractionated radiotherapy in various malignancies during the COVID-19 crisis, its role in intracavitary brachytherapy (ICBT) in cervical malignancy has been less emphasized. The present viewpoint thus recapitulates the practice of hypofractionated ICBT and nonsedative anesthesia techniques in patients with locally advanced cervical cancer (LACC) and discusses their potential role during the COVID-19 crisis. Although the viewpoint highlights on ICBT during the COVID-19 pandemic, it is not meant to replace fractionation schedule and anesthesia techniques being practiced.

## ICBT & its rationale during the crisis

Cervical cancer is the second leading cause of malignancy among the female population worldwide after carcinoma of the breast [3]. ICBT is an integral part of the management of cervical malignancy. In cervical cancer, it falls at priority level 1, having a very high (>50%) chance of successful curative treatment of rapidly proliferating tumors. The UK National Health Service rationalized management of cancer patients during the outbreak as per the treatment intent while continuing to deal with life-threatening emergencies, assessing the risk/benefit ratio to allow proper utilization of resources without overburdening the health system and suspending all non-urgent elective surgeries for 3 months starting from April 15th, 2020 [4] The ICBT procedure thus has potential utility and may be a prudent choice in light of the foreseeable reduction in surgical procedures amid the current pandemic crisis, where it might be used as a first-line treatment in early-stage cervical malignancies.

Furthermore, ICBT, along with external beam radiation therapy (EBRT), plays an important role in managing patients with LACC. As per the American Brachytherapy Society (ABS) recommendation, the OTT which includes (EBRT + ICBT) should be less than 8 weeks, beyond which local control and survival have been shown to decrease by ∼1% per day [5]. As treatment interruption due to the COVID-19 pandemic is bound to have a detrimental effect, ABS guidelines recommend that brachytherapy procedures for patients with cervical cancer should not be delayed in patients without the COVID-19 symptoms [6].

ICBT can also be interdigitated with EBRT in an attempt to reduce OTT. A study by Alam *et al.* concluded that interdigitated ICBT has equivalent response and toxicities to sequential ICBT, with the advantage of a significant reduction in OTT [7]. Also, by delivering an equivalent dose of EBRT with ICBT, it can ease the load on the EBRT facility by limiting the number of patient hospital visits and possible hospital exposure.

## Anesthesia in ICBT during crisis

ICBT is preferably done in the operating theater under GA for obtaining adequate pelvic floor relaxation, which allows the treating radiation oncologist to adequately pack and spare dose-limiting structures like the bladder and rectum. However, the shortage of anesthesia teams, lack of ventilators, and the risk of transmission of the infection during intubation for anesthesia has caused many centers worldwide to curtail their surgical procedures during the COVID-19 pandemic. Moreover, manpower shortage during the crisis has caused compromised inpatient services, affecting patients requiring a hospital stay for fractionated brachytherapy. Endotracheal intubation, an aerosol-generating procedure during GA, also risks the lives of the doctors; hence proper personal protective equipment and masks must be used throughout the procedure. If GA is still considered essential, a rapid sequence induction and intubation should be performed after preoxygenation for at least 5 min with 100% oxygen, as per recommendations [8]. A study by Yao *et al.* documented that rapid sequence induction or modified rapid sequence induction was used with an intubation success rate of 89.1% on the first attempt and 100% overall. Also, those critically ill patients who were intubated had a higher chance of developing hypoxemia and hypotension during and after the procedure [9]. Overall, ICBT applications under GA have been affected during the crisis phase.

Alternatively, ICBT done under conscious sedation is noninferior to GA as concluded in a study done by Sharma *et al.* [10] Thus, the use of conscious sedation for ICBT could be of help in reducing the need for GA during the COVID-19 crisis.

## Image-guided ICBT

Image-guided brachytherapy is performed worldwide. We have evolved over the decades from 2D orthogonal image-based ICBT to 3D computed tomography-based brachytherapy, along with the evolution of applicators and sources from radium to cobalt and iridium. It is known that MRI has a better accuracy to delineate the high-risk clinical target volume in LACC owing to its high soft-tissue resolution. However, MRI facilities as well as MRI-compatible applicators are not available in most centers because of the huge burden of disease, particularly in low- to medium-income countries.

With emerging evidence from the Groupe Européen de Curiethérapie and European Society for Radiotherapy and Oncology's retrospective European studies on MRI-guided brachytherapy in LACC (RetroEMBRACE and EMBRACE I), MRI-based adaptive brachytherapy has been proposed by the EMBRACE II study group [11]. MRI performed at each application or fraction with an applicator *in situ* is considered to be ideal but given the present scenario, MRI should be preserved only for those patients in whom it is expected to improve treatment delivery; hence the rationale to perform predominantly CT-based ICBT for patients with local disease or minimal parametrial invasion, with point A-based dose prescription [12].

## Dose & fractionation

Different brachytherapy fractionation schedules have also been listed as per ABS [12]. Brachytherapy dosage schedules vary from institute to institute, 7 Gy high dose rate each for four fractions being the standard recommendation. In a study done by Patel *et al.*, 9 Gy high dose rate each for two fractions showed comparable results with good local control and a minimum of normal tissue toxicity [13]. However, comparative International Atomic Energy Agency trial results favored the four-fraction schedule [14]. Although not a preferred brachytherapy schedule, weekly 9 Gy for two fractions, requiring less treatment time with negligible hospital stay, could be considered as an alternative option during the COVID-19 phase, especially in patients with low volume disease post-EBRT and in whom inferior local control, if at all, is acceptable. Otherwise, at least three fractions should be considered to achieve an equivalent dose in 2-Gy fractions (EQD2) greater than 85 Gy. A minimum gap of 6 h should also be maintained between the fractions.

## General measures

Besides general precautionary measures such as using sanitizers and wearing gloves, masks and other personal protective equipment, patients must be properly screened for COVID-19 related symptoms before being taken for the ICBT procedure. History of travel from a high-risk zone in the last 14 days as well as contact history with a known COVID-positive person should also be explored.

As most elective surgeries are being postponed because of the pandemic, the possible chance of cure in early-stage cervical cancer lies in the hands of the radiation oncologists. Thus, cases with early-stage cancer must be discussed in a multidisciplinary tumor board among gynecologic oncology surgeons and radiation oncologists, to arrive at a unanimous treatment decision. Also, treatment should be initiated without interruption whenever possible, minimizing the total treatment duration for maximum benefit.

## Patient concerns

The concerns of cancer patients should be kept in mind; many feel undertreated and inadequately addressed due to amendments in treatment protocols during the COVID-19 crisis. Also, because the outcomes of alternative cancer treatment protocols during the pandemic are currently unknown to most of the treating oncologists, a detailed discussion between the oncologists and patients should involve explaining their desired course of action and their expected outcomes. It should also be conveyed through effective communication that all decisions taken are in the best interest of the patient.

Although the fear of COVID-19 has engulfed the whole world, it is paramount for oncologists to stand by their patients during these tough times. Hence, instead of being panic-stricken, it is of utmost importance that we analyse the situation precisely and formulate treatment plans on an individual basis as deemed suitable. There is an essential component of empathy in routine oncology care that will continue behind the latest physical barriers of mask and telemedicine.

## Conclusions

Performing ICBT with the required minimum number of fractions after taking into consideration the EQD2 dose and acceptable late complications and utilizing anesthesia alternatives such as procedural sedation should be the aim during the COVID crisis. All proper safety, hygiene and screening must be ensured before each procedure. The concerns of patients should be prioritized and there should be thorough and effective communication regarding the best possible treatment and the probable outcomes.
